# Arsenic in Drinking Water in Bangladesh: Factors Affecting Child Health

**DOI:** 10.3389/fpubh.2014.00057

**Published:** 2014-06-16

**Authors:** Sonia N. Aziz, Khwaja M. S. Aziz, Kevin J. Boyle

**Affiliations:** ^1^Department of Economics, Moravian College, Bethlehem, PA, USA; ^2^Bangladesh Academy of Sciences, National Museum of Science and Technology Bhaban, Dhaka, Bangladesh; ^3^Department of Agricultural and Applied Economics, Virginia Tech, Blacksburg, VA, USA

**Keywords:** arsenic in drinking water, environmental economics, child health, empirical model, Bangladesh

## Abstract

The focus of this paper is to present an empirical model of factors affecting child health by observing actions households take to avoid exposure to arsenic in drinking water. Millions of Bangladeshis face multiple health hazards from high levels of arsenic in drinking water. Safe water sources are either expensive or difficult to access, affecting people’s individuals’ time available for work and ultimately affecting the health of household members. Since children are particularly susceptible and live with parents who are primary decision makers for sustenance, parental actions linking child health outcomes is used in the empirical model. Empirical results suggest that child health is significantly affected by the age and gender of the household water procurer. Adults with a high degree of concern for children’s health risk from arsenic contamination, and who actively mitigate their arsenic contaminated water have a positive effect on child health.

## Introduction

Widespread arsenic contamination of groundwater in Bangladesh places the health of millions of Bangladeshis in jeopardy while pathogen contaminated surface water serves as an alternative. Water sources without high arsenic levels or pathogen contamination are scarce, affecting peoples’ health. Children are particularly susceptible, putting them at a high level of ambient risk. Arsenic mitigation technologies are expensive, and water sources with low levels of arsenic may be few and far between, taking a toll on child health. People who wish to take actions to avoid arsenic exposure may not because mitigation requires a substantial commitment of resources and because the need for improved child health may be clouded by subjective perceptions of risk. The purpose of this study is to show how, in a developing country context, child health may be influenced by factors such as mitigation, time spent mitigating, parental productivity, demographic characteristics, and health perceptions of risk to children.

Though diseases caused by arsenic do not discriminate among individuals, children may have heightened susceptibilities to ambient arsenic hazards (1). People often make decisions to protect themselves and their families from environmental risk. A premium may be placed on protecting children in taking actions to reduce the risk, or to lessen the impacts of exposure to the risk. This paper is based on a theoretical note developed for parental and child health valuation (2), and on theoretical and empirical work on arsenic in drinking water in rural Bangladesh (3), and illustrates the empirical framework based on a mitigation model for adult behavioral responses to changes in perceived arsenic risk reductions to their children. Protective expenditures or actions that individuals undertake to avoid exposure to any undesirable outcome (e.g., pollution, illness, and death), reveal something about the value of avoiding environmental damage. This is based on the idea that the value of a small reduction in environmental risk can theoretically be estimated by the amount an individual is willing to sacrifice for any given mitigating action to prevent it. The valuation in this application is complicated by the heavy resource constraints faced by rural Bangladeshis. Most rural Bangladeshis do not have the luxury of choosing to pay for a convenient mitigation technology, but do wish to protect themselves and their children. In the context of arsenic contamination, this may mean walking farther to access water from a safe source. The nature of valuation for perceived risk reductions for people whose economic life is only partly or even slightly participative in a cash market economy may be expressed through investments in time rather than purchased inputs.

An additional complication with characterizing the protective decisions parents make to protect themselves and their children from risk involves the non-deterministic nature of the problem. In other words, how do we characterize the optimal mitigating choices parents wish to take when they do not know whether they or their households will become ill from arsenic exposure? We address this in the paper by using subjective (non-deterministic) notions of health risk from exposure to arsenic.

The theoretical questions and complications discussed above are motivated by the basic problem facing millions of Bangladeshis: tube wells that access groundwater are a primary source of drinking water in Bangladesh and allow households to avoid bacterial contamination of surface waters that lead to gastrointestinal diseases (4). In rural areas of Bangladesh tube wells have been painted green or red; green indicates that arsenic concentrations in the wells water is below the Bangladesh maximum contaminant level (mcl) of 0.05 mg/L and red indicates that the arsenic concentration is above this standard. The health message to local residents is that water from green wells is safe to drink and water from red wells is not safe to drink. This investigation is developed within a household production framework to access “safe” water from green tube wells. Because households can affect their exposure to arsenic through the drinking water choices they make, the model builds on the endogenous risk framework of Ehrlich and Becker (5); Quiggin (6); Nastis and Crocker (2); and Aziz (3). In this model, a representative household decision maker, who is the primary water procurer, is assumed to allocate household resources to maximize the utility of an altruistic parent, an assumption used in most research involving the economics of the family (7, 8). The objective in this paper is to employ an empirical model in which the health of the child is the dependent variable, which allows us to observe the effect of parental abilities, actions and perceptions on child health (9–11).

## Materials and Methods

This research uses an econometric specification similar to the one developed by Saha et al. (12) and Nastis and Crocker (2). The estimated model represents child health as the discrete ordered dependent variable, and is a function of risk, choosing to mitigate, parental characteristics (via work ability/productivity and physical characteristics).

The child health variable is discrete, like most health sector variables and its analysis requires non-linear estimation. Most discussions of ordinal variables are unsuitable to ordinary least squares (OLS) regression and structural equation methods. The reasons for this are these models do not explicitly recognize the ordinal nature of the variables, arbitrary assumptions about the distance between the ordered categories are inevitable and analysis of continuous, binary, and ordinal variables are difficult to discuss within a common statistical framework.

Ordered response models originated in the biometrics literature, and their appearance in the social sciences is attributed to McKelvey and Zavoina (13). Since then, many applications and extensions of these models have appeared in the economics literature. Keeping this in mind and following Greene (14), a paper by Liao (15) and Drichoutis et al. (16) guidelines on interpreting ordered response models, an econometrically sound ordered probit model was estimated. Please see Appendix B for details.

The empirical framework was modified to better fit the nature of the problem, we are estimating – the dependent variables is modeled as a discrete value, which is a common occurrence in health data. Appendix A details the mapping of survey variables and other data to the estimated equation. Several simplifying assumptions are made in order to appropriately specify the econometric system in accordance with the available data. Actual child health is a continuous variable. The indicator variable for child health is a measure of nutritional status commonly known as mid upper arm circumference (MUAC). The association between nutritional status and arsenicosis in Bangladesh studied by Milton et al. (17) add to previous evidence showing poor nutritional status may increase an individuals susceptibility to arsenicosis, or alternatively, positing arsenicosis may contribute to poor nutritional status. The observed data is MUAC in millimeters and so the actual available data is continuous. Two data limitation issues are of importance for MUAC.

First, since a child’s MUAC changes substantially with age, the MUAC was divided by age *a la* Almeida (18). Second, for the purposes of this analysis the indicator variable for child health is transformed into a discrete variable with lower ordered values indicating poor nutritional status while higher ordered values indicate better nutritional status. The reason for this is parents may not know the actual circumference of their child’s arm in millimeters but they are told by health workers whether their child is in poor health in four categories ranging from poor to good health. By extension, parent’s perceptions and decision-making is reflected by the discrete representation of the MUAC rather than its continuous counterpart.

The estimated equation presents child health via a discrete ordered probit. Equation 1 is the representation of the child health production function *h*^c^.

(1) $$h^{c}=\gamma_{r_{c}}^{c}r_{c}+\gamma_{z}^{c}z+\gamma_{\text{WA}}R_{\text{WA}}+\gamma_{\text{WM}}R_{\text{WM}}+\gamma_{r}^{c}\bar{r}+\gamma_{b_{c}}^{c}b_{c}+\varepsilon$$

where the ε is the econometric error term.

The child health function follows Saha et al. (12) and is a function of perceived arsenic risk for child (*r*_c_), the choice to mitigate (*z*), which corresponds to whether a household switches from a red to a green tubewell, parental productivity indicated by gains from mitigation via *R*_WA_ (increased work ability) and *R*_WM_ (increased work chances), the actual arsenic level in the tubewell water ($\bar{r}$) and physical characteristics of respondent (*b*_c_)^1^. Most of the indicators used for the independent variables are self-explanatory and direct (such as actual arsenic level in tubewell water). An indirect indicator of note is parental productivity as a result of mitigation, where subjective measures of productivity improvements are elicited via survey. It is assumed that improvements in the ability to work or increases in work opportunities, serves as a proxy for parental productivity.

## Data and Results

The study site and data collection were undertaken in the context of the International Center for Diarrheal Disease Research, Bangladesh (ICDDR,B’s) long term cross sectional and longitudinal research investigating health consequences of arsenic in drinking water in the Matlab area of rural Bangladesh (19). According to the British Geological Survey (20), southeastern Bangladesh, where Matlab is located, is the location in Bangladesh with the most pronounced arsenic contamination of shallow tubewell water. Tubewells that access groundwater are a major source of drinking water in Bangladesh (4).

### Data overview: Primary and secondary data

Existing data from an ongoing research initiative exploring health effects of arsenic exposure in the Matlab area are combined with primary data collected for this study. The Matlab area has seven study sub-divisions for ongoing research activities. This analysis used a stratified random sample of the population within the seven sub-divisions. Households were stratified based on the concentration of arsenic in the tubewell they currently use; with stratifications based on high (>50 μg/L), medium (25 to ≤50 μg/L), and low (<25 μg/L) levels of arsenic^2^. One thousand respondents were chosen per stratum for a target sample size of 3,000.

The primary data were collected through in-person interviews between March and June 2004 under the support of ICDDR,B. The survey was pre-tested by administering the instrument to 40 people outside of the sample area. The survey enumerator administered the survey to the member of the household that was identified as the primary water procurer. Health data are for this individual and the oldest child under 18 in the household. Enumerators were able to complete interviews with 2,800 households (21)^3^. About 610 of the survey respondents could not be linked to ICDDR,B’s existing data due to missing data in one or more of the existing secondary data sets so that the usable sample was reduced to 2,190 respondents. The survey collected demographic, health, and safety data on households, household sources of drinking and cooking water, and awareness of various issues related to arsenic.

The secondary data consists of other sets of exogenous household level data from the Health and Demographic Surveillance Systems (HDSS) database at ICDDR,B. The data sets available from the HDSS included data on indicators for child health such as immunizations for diphtheria, tetanus, polio, and measles as well as vitamin supplementation. The GIS component provided the data set describing drinking water history per household, including the level of arsenic in the tubewell currently in use – this provided the basis for stratifying the sample pollution and presented the objective level of arsenic hazard for both parent and child. The primary and secondary data sets were linked by unique respondent identification numbers.

### Descriptive statistics

The child health variable (*ChildHealth*) is constructed from the data as described in Table 1, and forms the dependent variable for the child health equation. *ChildHealth* is indicated by the nutritional status of the child; and is coded as one of four categories 1 through 4 where 1 represents acute malnutrition and 4 represents normal nutritional status: 57% of children had acute malnutrition. While this indicator of child health is not elicited as the parents’ subjective perception of their child’s health, health workers do inform parents of the health state corresponding to their child’s MUAC.

**Table 1:** **Descriptive statistics (*n* = 2,190)**.

Dependent variable	Definitions	Descriptive statistics
*ChildHealth* (*h*^c^)	1 = Acute malnutrition	57%
	2 = Severe malnutrition	38
	3 = Malnourished	0
	4 = Normal	5

Table 2 reports the independent variables. The personal interviews collected data for the variables *Avert* (*z*), *TimeAvert* (*w*), *WorkMore* (*R*_WM_), *WorkAbility* (*R*_WA_), *Age* (*b*_a_), and *Male* (*b*_m_), while the ICDDR,B secondary data provides the variables *ChildRisk* (*r*_c_) and *AAS* ($\bar{r}$).

**Table 2:** **Independent variables (*n* = 2,190)**.

Variables	Definitions	Descriptive statistics
*Avert* (*z*)	1 = Yes	34%
	0 = No	
*TimeAvert* (*w*)	Walking time to water source (minutes)	Mean = 44 (0–180)
*WorkMore* (*R*_WM_)	1 = No increase	23%
	2 = Little increase	7
	3 = Moderate increase	41
	4 = High increase	28
*WorkAbility* (*R*_WM_)	1 = No increase	23%
	2 = Little increase	7
	3 = Moderate increase	40
	4 = High increase	29
*ChildRisk*	Concerned about risk for child	
	1 = Yes	88%
	0 = No	12
*AAS* ($\bar{r}$)	Arsenic level (μg/L)	Mean = 227 (1–1,019)
*Age* (*b*_a_)	Age of respondent (years)	Mean = 43 14–106 years
*Male* (*b*_m_)	Sex of respondent (male)	22%

*Avert* represents a switch away from a red tubewell to a green tubewell, a safe drinking water source. About a third of respondents (34%) switched away from arsenic contaminated water in a red tubewell. Survey data revealed the prevalent mitigation method in the sampled set of respondents is switching to a green tubewell. Note that some respondents may not need to switch as the tubewell immediately adjacent to their home may be green. For respondents switching from a red tubewell to a green tubewell, *TimeAvert* reports the time spent for one trip to gather water from the green tubewell. The time ranged from 0 to 180 min with a mean of 44 min.

About a quarter (23%) of respondents did not report that averting arsenic improved their ability to work (*WorkAbility*) and their chances to work (*WorkMore*). Both of these are ordered categorical variables; respondents were asked to report their productivity changes from 1 through 4, where 1 corresponds to no chance or no increased ability to work, while 4 corresponds to a high increase in chance or ability to work.

*ChildRisk* is indicated by a binary variable corresponding to a survey question asking the respondent whether he or she is concerned about their child contracting health problems from arsenic in drinking water. Thirty-eight percent were concerned about getting sick from arsenic contaminated water.

*AAS* depicts the actual arsenic levels found in the tubewell. The arsenic levels are measured by field kit tests and subsequent laboratory tests carried out by ICDDR,B. The data shows that the average well had an arsenic concentration of 227 μg/L, which is nearly five times greater than the Bangladesh standard of 50 μg/L. The physical characteristics of the respondent are characterized by *Age* and *Male*, indicating gender of the survey respondent. The average age was 43 and 22% were male.

## Discussion

More than 50% of the explanatory variables are statistically significant in the estimated child health equation (Table 3). All significant variables appear to have the expected signs except *TimeAvert* and *WorkAbility* while the act of mitigation *Avert* appears to improve child health. This key result appears to indicate that while mitigation has a positive effect on child health, more time spent averting, and increases in work ability leaves household water procurers with less time and opportunity for necessary sustenance related activities for the household, including child care. It is important to note the value for TimeAvert is based on one trip to gather water from a water source safe from arsenic contamination reflecting an average one way trip time of 44 min. This suggests that health messages for households to avoid drinking arsenic contaminated water from red tubewells are having a behavioral effect on households despite its relatively high premium in time.

**Table 3:** **Coefficient estimates (*n* = 2,190)**.

Variables	*ChildHealth*
*Avert*	0.2711*** (0.1499)
*TimeAvert*	−0.00426** (0.0015)
*WorkMore*	0.0653 (0.0899)
*WorkAbility*	−0.2113** (0.0898)
*ChildRisk*	0.2998* (0.1302)
*AAS*	0.000327* (0.000326)
*Age*	0.0451*** (0.0042)
*Male*	−0.0656** (0.0322)
Log likelihood	
AIC	

^a^Significant at * <10%, ** <5%, *** <1%.

*^b^Standard errors are in parentheses*.

The positive effect of ChildRisk on child health suggests that subjective notions of improved health or health risk concerns for child is a significant factor affecting parental protective actions. Clearly a greater degree of concern for child risk from arsenic in drinking water directly affects people’s proclivity to take mitigating action, thereby affecting child health. It is interesting to note that age and gender significantly affect child health; household water procurers who are older and female have a positive effect on child health. All of the effects support the notion that villagers who perceive higher risks for their children from arsenic in drinking water take actions to reduce their exposure, which improves child health.

If parents perceive that children are particularly susceptible to arsenic in drinking water, public health directives on remediation of arsenic would purportedly have a strong effect on child health. Furthermore, public health awareness campaigns may benefit by focusing on socio-demographic characteristics highlighted by the results in this study. Previous work (22) also corroborates that caregivers who are older and female (usually the mother) are significantly more likely to be affected by awareness campaigns, therefore affecting actions taken to mitigate arsenic exposure both to themselves and to their children, unless other activities preclude them from doing so. A parent in rural Bangladesh, who typically has to care for many children, may not have the luxury of devoting much time or energy to take care of one sick child if they have the ability to pursue other household sustenance related activities. A future direction for work points to relative valuation of child versus parent health.

A stochastic decision-making model linking parent health and child health outcomes can be used to frame the relative valuation of child and parent health. Heavy resource constraints may influence relative valuation of child health over parent health. In relatively less resource constrained contexts, parents tend to value their child’s health higher than their own. Willingness to pay estimates from a study on parents’ valuation of latent health risks to their children show parents are willing to accept about a 2.5% point increase in risk of skin cancer to themselves in return for lowering this risk to their children by 1% point (9). Another study valuing health benefits of reducing environmental tobacco smoke exposure show smoking mothers on average value their child’s health roughly 1.5–1.7% points higher than their own health (23). These relative estimates for respondent parent and child health in conjunction with other studies suggest that at risk parents value their child’s health significantly higher than their own health (24, 25). The above studies corroborate the results in this paper showing parents value for child’s health. However, in contrast to the United States applications cited above, rural Bangladeshi children help provide the sustenance for the households. Rural Bangladeshi households also have more children on average than households in the United States. It would be interesting to note the relative value revealed by data from this study and to ask the degree to which parents value their child’s health more than their own when faced with heavy resource constraints.

Lastly, the empirical note in this paper, Eq. 1 acknowledges that mitigating actions may be constrained by time rather than by money in rural developing countries. Specifically, the impact of unpaid time and women as primary caregivers for children are important components of future research given the physical location and cultural context of this paper. Using appropriate estimates of unpaid time for women in rural agrarian developing economies is a crucial component in going forward with evaluation of factors affecting child health in rural Bangladesh.

## Conflict of Interest Statement

The authors declare that the research was conducted in the absence of any commercial or financial relationships that could be construed as a potential conflict of interest.

## Footnotes

^1^Note that *b_c_* indicates an index of physical characteristics in the theoretical model, in application this is indicated by age and gender.

^2^Stratification was based on ICDDR, B GIS data mapping tubewells currently in use. The arsenic levels are measured by field kit tests and subsequent laboratory tests (carried out by ICDDR,B).

^3^Each enumerator had access to the family health card allocated to each household in Matlab. A family health card has provisions to record detailed particulars of a family, including a unique identification number (RID) issued per household member, and includes information on number of living children, immunization status, health complications and record of services rendered to any family member by health service delivery sites.

## Appendix A

**Survey Questions used in this study:**

Avert (*z*_k_)

Question 47. Have you switched away from a tubewell due to arsenic contamination?

1 = Yes
0 = No

TimeAvert (*w*)

Question 52. From your home, how much time does it take to walk to and from the switched tubewell?

____________Minutes
____________Seconds

ChildRisk (*r*_m_)

Question 90. Are you concerned about your child getting sick from arsenic contaminated water?

1. Yes
2. No

WorkMore (*R*_WM_)

Question 71.3. Do you have increased work opportunities due to arsenic mitigation?

1 = No increase
2 = Little increase
3 = Moderate increase
4 = High increase

WorkAbility (*R*_WA_)

Question 71.3. Do you note increased ability to work due to arsenic mitigation?

1 = No increased ability
2 = Little increase
3 = Moderate increase
4 = High increase

AGE *b*^m^

MALE *b*^m^

Filled out by enumerator in survey header.

## Appendix B

The ordered probit model is built around a latent regression model similar in manner to the binary probit model (13). As in the binary probit model (which is the special case of *J* = 1), the mean and variance of ω is normalized to 0 and 1. Greene (14) specifies the following general model:

*y** = β′*x* + ω where the explained variable *y** is usually unobserved, β is the vector of parameters to be estimated and *x* is the vector of explanatory variables. Generally, *y* is observed with the following probabilities to be estimated, where the mean and variance of ω is normalized to 0 and 1.

$$\begin{array}{lll} \begin{array}{ccccc} y & =0 & \text{if} & y^{*}\leq 0 & \text{Prob (}y=\text{0)}=\phi \left(-\beta x\right)\\ y & =1 & \text{if} & 0\leq y^{*}\leq \mu_{\text{1}} & \begin{array}{c} \text{Prob (}y=\text{1)}=\phi \left(\mu_{\text{1}}-\beta x\right)\\ \;-\phi \left(\beta x\right) \end{array}\\ y & =2 & \text{if} & \mu_{\text{1}}\leq y^{*}\leq \mu_{2} & \begin{array}{c} \text{Prob (}y=\text{2)}=\phi \left(\mu_{2}-\beta x\right)\\ \;-\phi \left(\mu_{\text{1}}-\beta x\right) \end{array}\\ & \vdots & & & \\ y & =J & \text{if} & \mu_{J\text{-1}}\leq y^{*} & \text{Prob (}y=J\text{)}=1-\phi \left(\mu_{J-1}-\beta x\right) \end{array} & \; & \end{array}$$

In the general case, the μ*_J_*’s are unknown parameters to be estimated with β, and *y** unobserved. Since this research reports on a dependent variable from secondary data, the *y** is created given the appropriate cutoff value for μ*_J_* (e.g., ordered categories for mid upper arm circumference of the child).
